# Androstadienone sensitivity is associated with attention to emotions, social interactions, and sexual behavior in older U.S. adults

**DOI:** 10.1371/journal.pone.0280082

**Published:** 2023-01-13

**Authors:** David W. Kern, Gabriel T. Kaufmann, Tom A. Hummer, L. Philip Schumm, Kristen E. Wroblewski, Jayant M. Pinto, Martha K. McClintock

**Affiliations:** 1 Isidore Newman School, New Orleans, Louisiana, United States of America; 2 Pritzker School of Medicine, The University of Chicago, Chicago, Illinois, United States of America; 3 Department of Psychiatry, Indiana University School of Medicine, Indianapolis, Indiana, United States of America; 4 Department of Public Health Sciences, The University of Chicago, Chicago, Illinois, United States of America; 5 Department of Surgery, Section of Otolaryngology-Head and Neck Surgery, The University of Chicago, Chicago, Illinois, United States of America; 6 Department of Psychology, The Institute for Mind and Biology, The University of Chicago, Chicago, Illinois, United States of America; CHINA

## Abstract

Δ 4,16-androstadien-3-one (androstadienone) is a putative human pheromone often linked to sexual attraction in young adults, although specific associations with sexual behavior are not yet established. Androstadienone also serves a broader social-emotional function beyond the sexual domain, specifically tuning the brain to efficiently process emotional information. Whether these effects persist throughout the lifespan into post-reproductive life is unknown. In a laboratory study of older adults, those with greater androstadienone odor sensitivity paid greater attention to subliminal emotional information, specifically, angry faces (p = 0.05), with a similar relationship to happy faces. In contrast, the physical odor *n*-butanol (a control) did not affect emotional attention (p = 0.49). We then extended this laboratory research and determined whether sensitivity to androstadienone affects the everyday lives of older adults by measuring their social and sexual behavior. In this second study, we surveyed in a nationally representative sample of US older adults living in their homes (National Social Life and Aging Project, 62–90 years; n = 2,086), along with their sensitivity to androstadienone, general olfactory function, health and demographics. Greater sensitivity to androstadienone was associated with richer social lives: having more friends, increased communication with close friends and family, and more participation in organized social events and volunteer activities (all p’s ≤ 0.05, generalized linear models, adjusted for age and gender). It was also associated with more recent sexual activity, more frequent sexual thoughts, and viewing sex as an important part of life (all p’s ≤ 0.05). General olfactory function did not explain these associations, supporting a specialized function for this pheromone during everyday life, and expanding its role to social life as well as sexual behavior, likely mediated by enhanced attention to emotional information.

## Introduction

Social interaction plays a vital role in health across a wide variety of taxa and in many cases such interactions are affected by chemical communication. In both insects and mammals, pheromones trigger specific social and sexual behaviors [1,2]. In contrast, human chemosignals modulate behavior in relatively subtle ways and do so primarily in a social context [2–7]. One such example is the putative human pheromone Δ4,16-androstadien-3-one (androstadienone), which is derived from progesterone and has been identified in human plasma [8], sweat [9,10], axillary hair [11], and semen [12]. Although detectable as an odor by some, like other body scents with biological activity, androstadienone’s odor is not typically associated with people [13,14]. and is effective in nanomolar amounts well below conscious detection [15].

Androstadienone is detected at varying concentrations by different individuals, which is similar to many other odors and pheromones; furthermore, it may function as a subliminal odor, or vasana, which operates below the threshold of conscious odor detection [3]. It is known that the activity of olfactory receptor neurons correlates with odorant concentration across a variety of odorants in *Drosophila* models [16], suggesting a common set of olfactory receptors leading to these differences in androstadienone sensitivity, contributing to its varying role as a pheromone, vasana, or odor. Furthermore, mRNA expression has been shown to change in response to odor concentrations [17], suggesting further that a common set of olfactory receptors for androstadienone may mediate its varying conscious and subconscious effects.

Androstadienone has been conceptualized as a modulator of sexual attraction, particularly by fragrance makers, although to our knowledge, there are no extant data demonstrating its association with sexual behavior per se, either in the laboratory or in everyday life. Rather, the extant literature focuses on adults of reproductive age and the sexual attractiveness of images, sensual mood, and differences in brain processing between men and women of different sexual orientations, as well as more recently, voice modulation, with varying results [4,6,18–23]. However, its effects are not always sex-specific [4,24–27].

We have hypothesized that androstadienone has a broader social function. It heightens attention to social emotional stimuli (happy and angry faces), as well as emotional words [28]. It modulates mood in the laboratory and in everyday life, increasing positive moods, attenuating negative ones and heightening a sense of attention and focus [29]. Specifically, subliminal androstadienone modulates the distributed neural systems involved in emotion and attention (in Positron Emission Tomography) [5]. The amygdala is the primary driver of its effects on prefrontal and orbital frontal cortices and attentional systems exert top-down control of emotional processing, but only when people were looking at emotional images (in dynamic causal modeling of fMRI imaging) [25]. These psychological and neurological findings support the theory that androstadienone serves a broader social emotional function by tuning an individual’s attention toward emotionally salient information, including social information, beyond a narrow role in sexual behavior.

To extend our socio-emotional hypothesis beyond a reproductive context, we studied older post-reproductive adults to determine whether androstadienone sensitivity is positively associated with attention to subliminal emotions and with their social and sexual behaviors during everyday life, extending the laboratory research into the field. In Study 1, we conducted a laboratory study of older adults to determine (1) whether androstadienone modulates their attention to subliminal emotional stimuli as it does in young adults, and (2) the relationships between this attentional bias and their sensitivity to the odors of androstadienone and of *n*-butanol, a physical control odorant.

Study 2 tested our hypothesis that androstadienone operates during the everyday social and sexual lives of older adults. We conducted a large representative field survey of older US adults living in their homes (National Social Life, Health, and Aging Project, NSHAP). Using a person’s ability to detect the odor of androstadienone as a biomarker or proxy for its effects on emotional processing, we first established national norms for the diverse US population and quantified the full range of individual differences in sensitivity to androstadienone. In addition to this social odor, we also measured sensitivity to *n*-butanol, a physical odor, and odor identification, a task requiring not only odor detection but also the memory, language and visuo-spatial abilities required to identify the odor. Finally, we asked detailed survey questions about the older adults’ social and sexual activities and tested their associations with androstadienone sensitivity. These two studies each have their own Introduction, Methods and Results and are then synthesized in a single General Discussion.

## Study 1: Androstadienone enhances attention of older adults to subliminal emotional faces

### Introduction

Study 1 was a controlled laboratory experiment with older adults to determine whether androstadienone increases their attention to subliminal emotional information as it does in young adults [24,25]. It also tested our working hypothesis that people who respond to androstadienone with enhanced attention towards subliminal emotions in the laboratory are also more sensitive to its odor. Androstadienone odor sensitivity is a proxy for the presence of androstadienone olfactory receptor alleles that presumably also mediate effects of subliminal concentrations of androstadienone [30]. This lays the foundation for interpreting individual differences in androstadienone odor sensitivity in the US population of older adults and their associations with social and sexual behavior during everyday life (Study 2).

### Methods

#### Participants

Twenty older adults (57–77 years of age [12 women]) were recruited through advertisements posted on the University of Chicago campus (criteria:≥ 57 years of age, English speakers, cognitively capable of participation and normal corrected vision. No volunteers were excluded). This study was approved by the University of Chicago Social Sciences Division Institutional Review Board; all participants provided written, informed consent and were paid $10 upon completion of the study.

Testing took place in a private, well-ventilated room designed for olfactory testing with 100% exhaust [28]. Participants were not permitted to consume any food or beverage other than water during the 30 minutes before testing [31].

#### Odorant preparation

Androstadienone (Steraloids, Inc., Newport, RI) solutions were prepared using established protocols [29,32,33]. Here, 16 different pens (Sniffin’ Sticks; Burghart Messtechnik, Wedel, Germany) containing androstadienone solutions were created by dissolving a stock solution into 16 dilutions with a 2-fold increase of liquid concentration, ranging from 3000 μM (dilution 1) to 0.091 μM (dilution 16). Two control pens contained only the carrier solution.

*N*-butanol sensitivity, a standard physical odorant, served as an indicator of general olfactory function, also presented with Sniffin’ Sticks in a validated olfactory threshold test [34]. Sixteen *n-*butanol solution pens, identical to those prepared for androstadienone, ranged in concentration from 3000 μM (dilution/pen 1) to 0.091 μM (dilution/pen 16; Burghart Messtechnik, Wedel, Germany). Again, two control pens contained only the carrier solution.

For each odorant, respondents were presented with a pen that contained the highest concentration that would be presented (androstadienone 5.3% [3000 μM], *n*-butanol 3000 μM [dilution/pen 1]) This essential component of psychophysical testing ensured that respondents first knew what stimulus they would be asked to detect before the test began [31,35]. Respondents were asked if they were able to smell the odor and told explicitly that this is the target odor they would be attempting to detect during testing.

#### Olfactory sensitivity: Staircase method

Odor sensitivity was determined for both androstadienone and *n*-butanol using the classic full staircase protocol [31,36]. This test also controlled the participants’ odorant exposure. Each test began with one of the two weakest dilutions (pen 15 or 16), chosen randomly. During each presentation, three felt tip pens were offered to the participant: one pen contained the target odor while the other two contained only the carrier solution. Relative position of the target pen in the sequence of three pens rotated with each presentation. Participants indicated which of the three pens contained the target odor. If at any point the participant gave an incorrect response, the test was made easier and a stronger concentration (i.e., less diluted) that was twice the strength of the previous was presented. After a correct answer at a given dilution, that same dilution was presented a second time in case the original correct response was due to chance alone (as expected one third of the time). Following two consecutive correct responses at a given dilution, the test was made more difficult by presenting a weaker concentration (greater dilution) that was half the strength of the previous one. Each change from weaker to stronger or stronger to weaker was considered a “reversal.” The staircase protocol was administered until 7 reversals were observed. The dilutions presented at each of the last 4 reversals were then averaged to estimate that participant’s odor threshold [31,37].

#### Vision

Older adult participants might have poor vision that would impair their ability to unconsciously perceive subliminal visual stimuli in the emotional bias attention task. We ensured adequate visual acuity in two ways. First, participants wore their habitual eye correction (glasses or contacts) for visual testing and throughout the experiment. Prior to olfactory testing, near visual acuity was measured (Sloan letter near vision card at 40 cm) and all participants had a near visual acuity of at least 20/32 with correction. Secondly, we increased the size of the face stimuli from 56 x 66 mm to 70 x 80 mm as has been used previously for testing older adults with the dot-probe task [38].

#### Unconscious emotional attention: Dot probe task

The strength of biased attention towards emotional stimuli was quantified with the well-established visual “dot probe” task previously used to assess behavioral changes with exposure to androstadienone in young adults [28]. The dot probe task subliminally displays an emotional face paired with a neutral face, on either the left or the right side of a screen. The emotional stimulus unconsciously shifts participants’ attention to the left or right towards the emotional stimulus and away from the neutral stimulus. Prior to presentation of subliminal faces, participants fixated on a cross in the middle of the screen for a random amount of time ranging from 500 ms to 1000 ms. Two faces of the same individual were presented subliminally for 27 ms on either side of the fixation cross, followed by scrambled faces for 67 ms to ensure that any awareness to the subliminal stimuli was masked [39]. Such masking techniques eliminate conscious awareness of the emotional stimulus. For example, masking reverses the attention paid to emotional faces in those with social anxiety [40,41]. Omitting masking and using long presentation times resulted in failure to detect the biasing effect of androstadienone on emotional faces while its effect on an emotional Stroop test was replicated [42].

The dot probe (an asterisk) then appeared on either the left or right side of the screen. For each trial, the person had either a happy or angry expression and the other was neutral, or both faces were neutral. Each participant was instructed to fixate on the cross in the center of the screen, and then press a button to indicate as quickly as possible whether the dot probe (asterisk) appeared on the left or right (Z key for left, M key for right).Faces were male and female Japanese and White faces that have been utilized previously to assess recognition of emotional expression [43] and were comprised of the same set of stimuli used previously with similar methods [28]. The dot probe task was presented via E-prime® software (Psychology Software Tools, Inc., Pittsburgh, PA).

During trials in which the dot probe appears on the same side as an emotional face, matching trials, response speed is faster because the participant has already attended to the emotional information (i.e., the participant is already looking in the direction of the emotional face). Trials during which the probe appears opposite the emotional face, non-matching trials, result in slower response times because the participant is attending to the emotional face and requires more time to find the dot probe (i.e., the participant must shift their attention away from the emotional face and find the dot probe on the opposite side of the screen). Following standard protocol, an emotional attention bias score for each participant was calculated as the difference between the mean reaction time from non-matching trials (i.e., the dot probe was located contralateral to the emotional face) and the mean reaction time for matching trials (i.e., the dot probe was located ipsilateral to the emotional face) [44]. Higher emotional attention bias scores indicate greater attention paid to emotional information wherever their location.

If androstadienone increases attention specifically to emotional faces (the probes), reaction times should be both faster in response to ipsilateral positions and slower to contralateral position. If androstadienone merely increases overall attention, reaction times should be equally fast on all trials regardless of probe location.

#### Laboratory protocol and design

After providing informed consent, demographic information, and vision measurement, participants practiced ten dot-probe trials to ensure they understood the procedure. Because people differ significantly in attention to emotional information, the strength of each participant’s emotional attention bias was established in a Baseline Block of 100 dot probe trials. After odorant exposure, the Odorant Block of 100 dot probe trials measured the change from baseline in emotional bias within subjects. The effects of androstadienone and *n*-butanol exposure were compared with a between subjects randomly assigned design: Androstadienone Condition (N = 10) and the *N*-butanol Condition (N = 10).

In the Androstadienone Condition, the androstadienone staircase threshold test exposed participants to androstadienone prior to the Odor Block dot-probe trials, and in the *N*-butanol Condition, the *n*-butanol threshold staircase test was administered. Because the staircase protocol is equally long in the two odorant conditions, this controlled for duration of odor exposure and the interval between the Baseline and Odorant Blocks of dot probe trials.

To measure olfactory sensitivity to both odorants in all participants, those in the Androstadienone Condition completed the *n*-butanol threshold test after their Odor Block dot probe trials and those in the *N*-butanol Condition completed the androstadienone threshold test.

#### Statistical analysis

Only reaction time data from accurate responses were used in analysis (98.6% ± 0.4% accuracy), following the conventional protocol [28,39,44]. Unusually long reaction times were removed as they reflect inattention to the task [45]; > 2 times the participant’s interquartile range (3.2% of accurate responses). Very short reaction times < 200 ms were also removed, as they likely result from accidental or over-anticipatory button-pressing. One participant was uneasy with computer use, was only 52% accurate, and excluded from dot probe analyses.

The within-person design enabled repeated measures ANOVAs to compare emotional bias scores in the Baseline and Odorant Blocks, with odor exposure (baseline, odorant) and face valence (happy, angry) as within-person factors. Separate ANOVAs were conducted for the Androstadienone and *N*-butanol Conditions. Model assumptions were verified using residual versus fitted plots, Q-Q plots, and histograms. A sensitivity analysis was performed, that confirmed the ANOVA findings, by fitting mixed-effects linear regression models with a Kenward-Roger small sample adjustment (data not shown) (Kenward and Roger, 1997). These models included odor exposure (baseline, odorant), face valence (happy, angry), and their interaction as independent variables with a random effect for participant. Odorant effects on overall reaction time was also tested with repeated measures ANOVA, utilizing only neutral-neutral paired stimuli. Lastly, the association between the magnitude of change in emotional bias and odor sensitivity was tested with a Pearson product-moment correlation, separately for each odorant (androstadienone and *n*-butanol) and for each valence of an emotional face (happy, angry). The association between the participants’ thresholds for androstadienone and *n*-butanol was tested with a Pearson product-moment correlation.

### Results

Average thresholds indicated that older adults, like young adults, are less sensitive to androstadienone than to *n*-butanol, the physical odor control (androstadienone sensitivity = 3.69 ± 0.55 dilution steps vs. *n*-butanol sensitivity = 7.4 ± 0.60 dilution steps). The distributions of sensitivity were also not the same. Fully 50% of participants could not detect androstadienone at all (i.e., had thresholds ≤ 2 dilution steps), creating a bimodal distribution which contrasted sharply with the *n*-butanol distribution in which 100% of participants detected *n*-butanol, demonstrating much broader individual variation in androstadienone than in *n*-butanol sensitivity. Among those who could detect androstadienone, the mean sensitivity was 4.25 ± 1.17 dilution steps, corresponding approximately to a 0.5% solution. Sensitivity to androstadienone and *n*-butanol were not significantly associated (*r* = -0.08, p = 0.74), as reported in previous laboratory studies of young adults [25,29].

**Fig 1:**
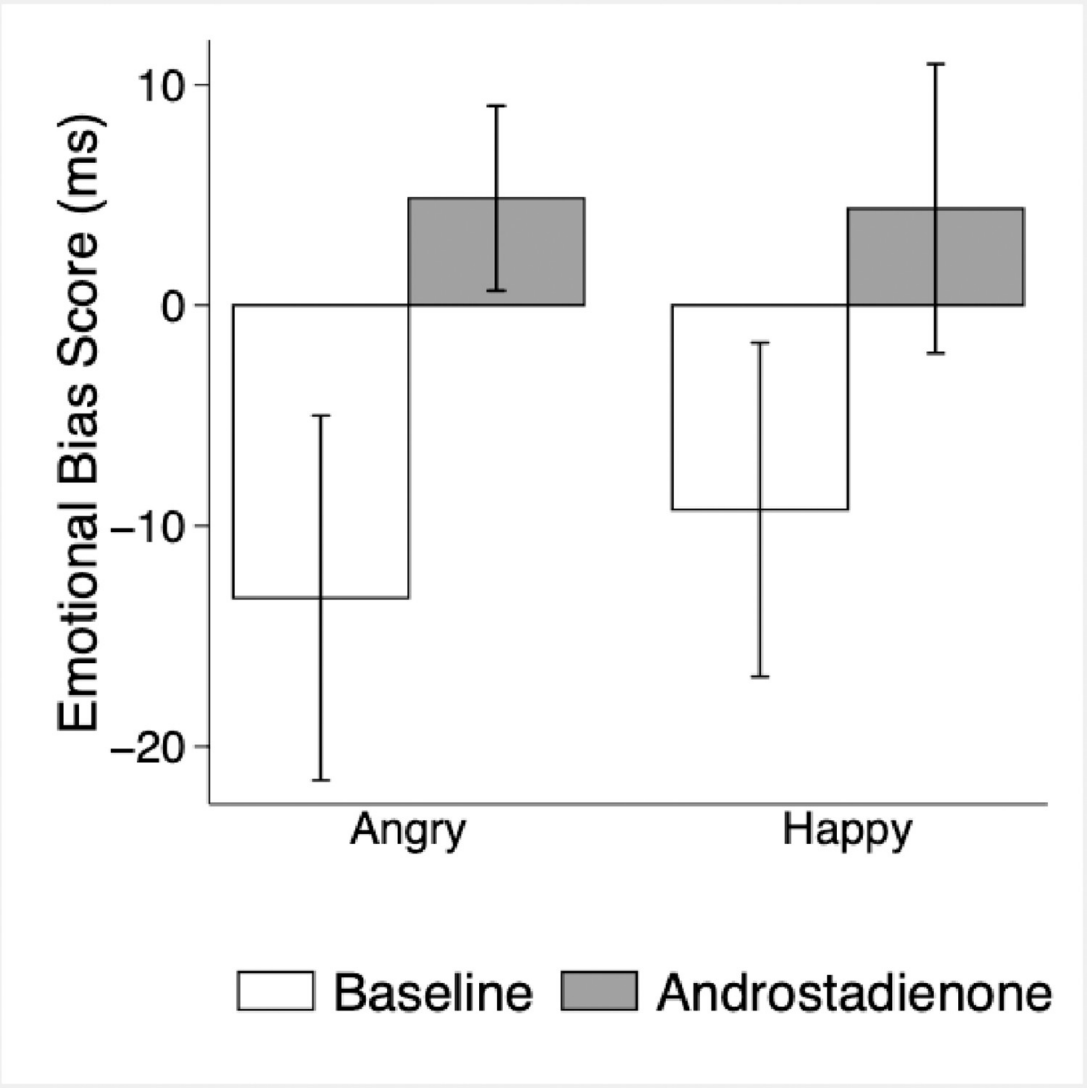
Emotional attention bias to angry and happy faces (mean ± SEM dot probe task scores) at baseline and after exposure to androstadienone. Higher emotional bias scores indicate greater attention to emotional faces.

**Fig 2:**
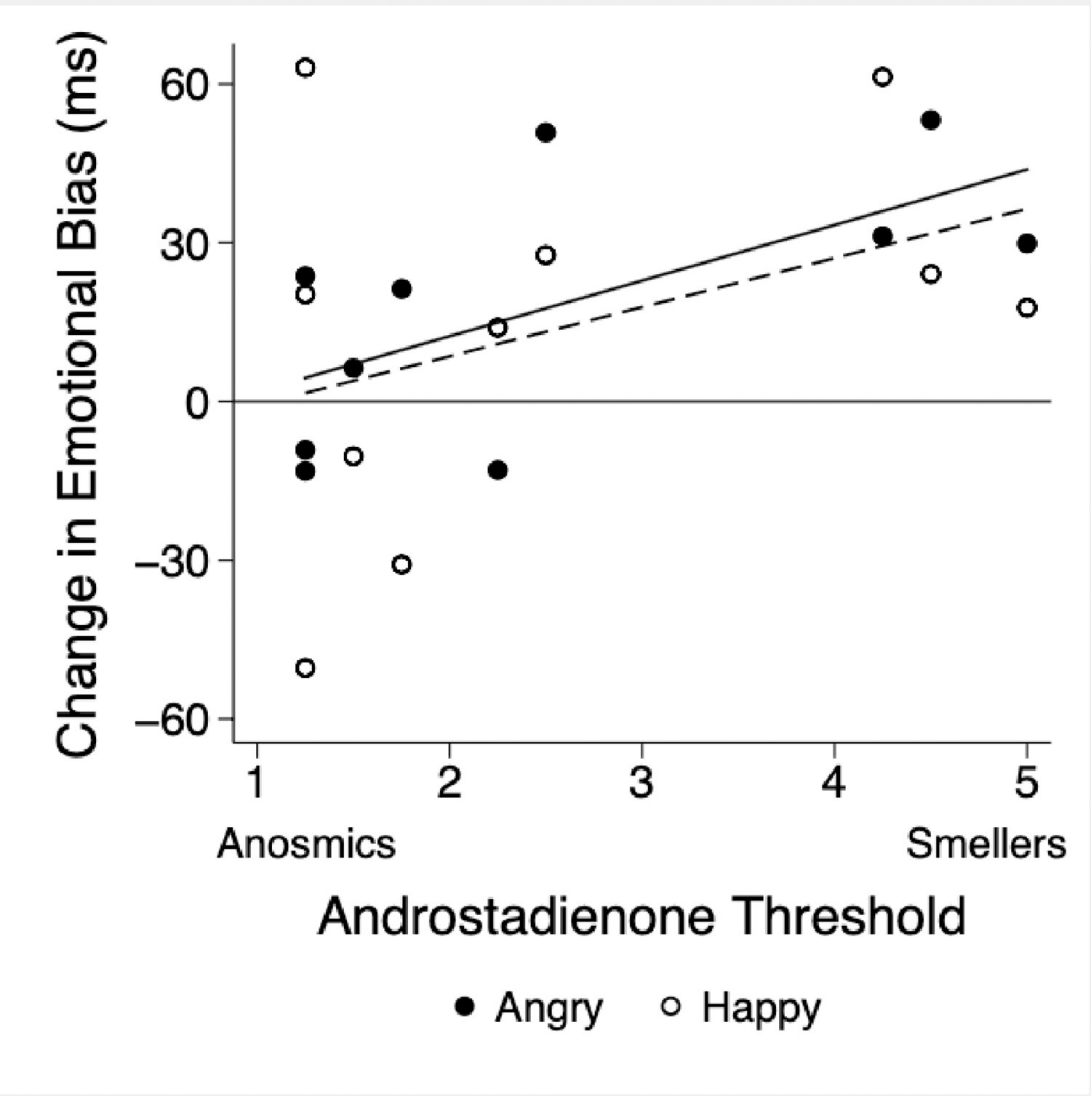
Androstadienone threshold (dilution steps; 3000 μM (dilution 1) to 0.091 μM (dilution 16)) and effect of androstadienone exposure on change in biased attention toward angry (r = 0.63; p = 0.05, solid regression line) and happy (r = 0.38; p = 0.28, dashed regression line) faces. There was no interaction with emotional valence of the faces (p = 0.69).

Androstadienone showed a trend toward increasing the attention of older adults to subliminal emotional faces, particularly angry ones (Fig 1, overall trend p = 0.08) as it had in younger adults [24]. Those older adults who responded to androstadienone with the strongest biases towards angry faces were also the most sensitive to its odor (Fig 2, *r* = 0.63, p = 0.05; a positive correlation between enhancing an emotional bias towards angry faces and being able to detect androstadienone’s odor at weaker concentrations). A similar relationship was observed for happy faces (i.e., no significant interaction with emotional valence, p = 0.69), although it alone was not statistically significant (*r* = 0.38, p = 0.28), likely due to the one anosmic participant who evidenced a strong emotional bias to happy faces (Fig 2). Indeed, 100% of participants who could detect the odor of androstadienone also responded to it with an emotional bias towards both angry and happy faces. Thus, in the field survey (Study 2) we could interpret androstadienone odor sensitivity as a proxy for its effects on emotional processing.

The effect of biasing attention was specific to processing emotional faces. Androstadienone did not shorten reaction times overall during the dot-probe task with neutral face pairs (p = 0.35) or alter the significant practice effect between Baseline and Olfactory blocks; there was a practice effect (p<0.01), but this did not vary by androstadienone exposure, indicating that it did not have a general effect on overall attention or sensorimotor processing speed.

As expected, general olfactory function did not mediate androstadienone’s effects. *N*-butanol did not affect attentional bias to emotional faces (p = 0.49) nor was there was an association between *n*-butanol odor sensitivity and change in emotional bias for either angry faces (p = 0.99) or happy faces (p = 0.97).

The staircase threshold procedure for measuring sensitivity and exposing participants to androstadienone is dynamic; the number of stimuli presented, and the strength of the dilutions are dependent on a given participant’s sensitivity and ability to accurately detect each presentation. As a result, not all participants were exposed to the same amount of androstadienone. One may hypothesize that longer exposure to androstadienone may have resulted in greater emotional bias. However, in the extreme case of selective anosmia for androstadienone, in which participants were necessarily exposed to the strongest concentrations because of the staircase method, these selective anosmics still showed less emotional bias than the most sensitive participants (who were exposed to weaker concentrations and therefore less androstadienone in total). Therefore, it is unlikely that any differences in exposure explain our results. Rather, they reflect a conservative estimate of the effect of androstadienone on the most sensitive individuals.

## Study 2: Association between androstadienone sensitivity and social and sexual behavior of older U.S. adults

### Introduction

If variation in androstadienone sensitivity is associated with social behavior as well as sexuality during the everyday lives of older adults, it would demonstrate a social function for this human chemosignal that is broader than sexuality alone, particularly if detected among post-reproductive older adults. Here we focus on socializing with friends and the community as well as sexual behavior.

By moving from the laboratory into the field, we sought to quantify the degree of individual variation in androstadienone sensitivity that could in turn be associated with differences in social and sexual behavior. Androstadienone odor sensitivity served as a proxy or biomarker for its modulatory effect on emotional processing. To establish national norms for androstadienone sensitivity in the diverse US population, The National Social Life Health and Aging Project (NSHAP) surveyed three aspects of olfactory function in a large representative sample of US older adults living at home: androstadienone sensitivity, *n*-butanol sensitivity, and odor identification [32](Kern, Wroblewski, Schumm, Pinto, Chen, et al., 2014). NSHAP is designed to statistically represent all home-dwelling U.S. adults and thus reflect its diverse population [46,47]. All respondents were in the post-reproductive phase of their lifespan (62–90 years of age).

### Methods

#### Respondents

NSHAP surveyed olfactory function in 2,180 older adults and their partners, ages 62–90 years in 2010–11 (a designed random subsample that was approximately two-thirds of all in Round 2). The University of Chicago Institutional Review Board approval was waived, as all data was completely deidentified prior to analysis. Professional interviewers from NORC at the University of Chicago used Sniffin’ Sticks (Burghart Messtechnik, Wedel, Germany) in a survey adapted protocol as part of a two-hour interview that took place in respondents’ homes and included questions about participants’ social life, sexual behavior, health, cognition and demographics (Table 1). A total of 2,086 respondents consented and completed an androstadienone sensitivity test, of which 2,083 also completed the *n*-butanol sensitivity test, and 2,085 completed an odor identification test [32].

**Table 1:** Distributions of olfactory function (# correct), demographics, cognitive function and health of older US adults living at home (NB: Tables are large and at the end of the manuscript).

	Sample	US Population	
	N	%	Mean ± SD
Androstadienone sensitivity	2,086		
0		38.6	
1		24.4	
2		21.5	
3		13.5	
4		2.0	
*n-*Butanol sensitivity	2,083		
0		8.8	
1		7.8	
2		12.0	
3		17.8	
4		25.1	
5		20.4	
6		8.1	
Odor identification	2,085		
0		2.8	
1		2.7	
2		6.1	
3		10.8	
4		32.2	
5		45.4	
Age	2,086		72.4 ± 7.5
Gender	2,086		
Men		46.8	
Women		53.3	
Race/Ethnicity	2,078		
White		81.5	
African American		9.8	
Hispanic		6.3	
Other		2.4	
Cognition (MoCA-SA)	2,086		14.0 ± 3.9
Self-rated physical health	2,083		3.3 ± 1.1
Self-rated mental health	2,085		3.7 ± 1.0

Detailed survey design, data collection, and baseline characteristics of the study population are available [46,48,49]. The data described here are publicly accessible through the National Archive of Computerized Data on Aging (NACDA, https://www.icpsr.umich.edu/icpsrweb/NACDA/). The Institutional Review Boards of The University of Chicago and NORC approved this study and written informed consent was obtained from all respondents.

#### Androstadienone sensitivity

As in Study 1, androstadienone (Steraloids, Inc., Newport, RI) solutions were prepared dissolved in propylene glycol using established protocols for presentation in Sniffin’ Sticks pens [29,32,33], Four ascending concentrations: 0.13% [93 uM], 0.5% [375 uM], 2.0% [1500 uM] and 5.3% [4000 uM] were selected to cover the full range of individual differences in sensitivity, including those who could not detect androstadienone even at very high concentrations [29,32,33].

Respondents were first presented with a target pen with the highest concentration of androstadienone (5.3% [4000 μM]) and asked if they could detect it. This established the target odor before they were actually asked to distinguish it from two control pens containing the carrier alone [31,35].

To reduce frustration and maintain cooperation throughout the rest of the survey interview, respondents who could not smell the androstadienone target pen did not continue androstadienone sensitivity testing. They were designated anosmic for androstadienone, recognizing that failure to detect androstadienone odor is common, dependent on olfactory receptor gene OR7D4 alleles, and not a product of general olfactory acuity [29].

Respondents who could smell the androstadienone target pen, or were unsure, were administered the sensitivity task using the four ascending concentrations of androstadienone. As in the staircase and constant stimuli methods [50,51], participants were presented in each trial with three Sniffin’ Sticks (2 blanks containing only propylene glycol and 1 target containing a given concentration of androstadienone dissolved in propylene glycol) and asked to select which of the three contained the target odor.

The number of androstadienone pens correctly detected (0–4) was analyzed as a continuous variable, with a response of “Don’t know” or a refusal to detect any of the items scored as incorrect. Those respondents who indicated they could not smell androstadienone in the target pen or were unsure were assigned a 0 score. The four concentrations were selected to ensure that these scores correlated closely with those from staircase-based methods [50].

#### *N*-butanol sensitivity

*N*-butanol sensitivity, a standard physical odorant, served as an indicator of general olfactory function and was also measured using Sniffin’ Sticks in a validated olfactory function test. Six odor concentrations were selected to cover the range of olfactory dysfunction in the home-dwelling population of older U.S. adults [50,52]: 0.13% (93.75 uM), 0.25% (187.5 uM), 0.5% (375 uM), 2.0% (1500 uM), 4.0% (3000 uM), and 8.0% (6000 uM). The test has excellent reliability [50], and followed the protocol of the androstadienone sensitivity task. *N*-butanol sensitivity scores ranged from (0–6). A response of “Don’t know” or a refusal was scored as incorrect. All respondents received the full *n*-butanol sensitivity test regardless of their stated ability to detect the target pen odor (8.0%) as there is no known genetic variation underlying anosmia to *n*-butanol odor. Only data from participants with androstadienone data are presented here.

#### Odor identification

The Sniffin’ Sticks odor identification test also served as an indicator of olfaction function. It requires respondents to identify each odor by choosing from a set of four word/picture choices [53,54]. The target odors were rose, leather, orange, fish, and peppermint, [33,55]). Item response analysis verified that odor identification scores ranged from (0–5; [53,54]. Again, responses of “Don’t know” or refusal were scored as incorrect. Using previously validated cutoffs for the odor identification task, the number of correctly identified odors (0–5) was categorized as normosmic = 5–4 correct and dysfunction ≤ 3 correct [33,54]. Only data from participants with androstadienone data are presented here.

#### Social and sexual behaviors

NSHAP asked detailed questions about social lives (Table 2): number of friends, number of close friends/family with whom they spoke at least once a year, how often they socialized with friends/family, how often they attended organized events and how often they volunteered [56,57]. NSHAP also assessed frequency of sexual activity, how often respondents thought about sex, how important they thought sex was as part of their life and whether or not they had an intimate partner (e.g. married or living with a romantic partner; [58–60].

**Table 2:** Distributions of social life and sexual behavior among older US adults living at home. (NB Tables are large and at the end of the manuscript).

	Sample	US		Sample	US
	N	%		N	%
How many friends do you have?	2,046		Have you had sex in the last 3 months?	2,086	
none		2.2	Yes		37.6
one		3.2	No		62.4
2–3		16.0			
4–9		32.0			
10–20		24.4			
More than 20		22.2			
How many close friends/family do you talk to at least once a year?	2,081		How often do you think about sex?	1,990	
0		0.0	Never		13.9
1		4.6	Less than once a month		23.6
2		9.1	One to a few times a month		24.7
3		14.5	One to a few times a week		23.4
4		17.3	Every day		11.5
5		24.5	Several times a day		2.9
6		25.6			
7		4.5			
How often did you socialize with friends/family in the past year?	1,821		How important is sex?	1,746	
Never		1.5	Not at all important		35.0
Less than once a year		1.2	Somewhat important		20.0
About once or twice a year		5.2	Moderately important		22.8
Several times a year		16.5	Very important		16.5
About once a month		22.4	Extremely important		5.8
Every week		36.2			
Several times a week		17.1			
How often have you attended meetings or organized groups in the past year?	1,813				
Never		29.4	Do you have an intimate partner?	2,086	
Less than once a year		7.1	Yes		70.4
About once or twice a year		8.6	No		29.6
Several times a year		9.3			
About once a month		17.6			
Every week		17.9			
Several times a week		10.3			
How often did you volunteer in the past year?	1,815				
Never		36.1			
Less than once a year		8.9			
About once or twice a year		11.8			
Several times a year		10.9			
About once a month		10.3			
Every week		14.7			
Several times a week		7.2			

#### Potential confounders

Race/ethnicity, worse cognitive function, or poor physical or mental health may have impaired a respondent’s ability to perform the sensitivity tests and may also reduce the frequency of social and sexual behaviors, creating spurious associations [55,61,62]. Race/ethnicity was categorized using the National Institutes of Health classification standards. Cognitive function was measured with the survey adapted Montreal Cognitive Assessment [63–65]. Respondents rated their physical and mental health on a five-point scale ranging from poor (1) to excellent (5), which are meaningful indicators of health and mortality [66] and with standard measures of depression and anxiety [67].

#### Statistical analysis

Estimates of prevalence and mean values in the U.S. population of home-dwelling older adults were based on weights adjusting for differential probabilities of selection and non-response. Design-based standard errors were determined using the linearization method [68] and the strata and Primary Sampling Unit (PSU) indicator are available with the NSHAP public dataset [46]. In contrast to previous epidemiological studies of olfaction, which have drawn from populations in limited geographical areas [69,70], the sampling design employed by NSHAP ensures that it is representative of home-dwelling older US adults between the ages of 62 and 90 years old.

Linear regression models tested gender and age differences in androstadienone sensitivity. Pearson correlation coefficients assessed the relationship between androstadienone sensitivity and general olfactory function measured by sensitivity to *n*-butanol and odor identification. No adjustments for multiple comparisons were made.

The association of androstadienone sensitivity with social and sexual activities was estimated using generalized linear models adjusting for age and gender. Coefficients were standardized (mean = 0 and SD = 1) for ease of interpretability. As a result, coefficients can be interpreted as the correlation between androstadienone score and a given dependent variable adjusted for age and gender. To ensure that any associations between androstadienone sensitivity and social or sexual behaviors were not affected by olfactory dysfunction more broadly, a possibility raised by the association between androstadienone sensitivity and olfactory function among older adults, confirmatory analyses were conducted by re-estimating all associations using the subset of the population who were classified as normosmic on the odor identification task.

Each potential confounding variable was first tested for an association with sensitivity to androstadienone separately using linear regression (race/ethnicity, cognition, physical and mental health). Those potential confounders that were significantly associated with ability to detect androstadienone were then separately added to the significant regression models testing the association of androstadienone sensitivity and social and sexual variables among the best smellers as demonstrated by the odor identification task.

All statistical analyses were conducted with Stata Version 13.1 (Stata Corp LP, College Station, Texas, USA). Many exact p values were ≤ 10−7 but are reported as p< 0.001 throughout for clarity.

### Results

#### Androstadiene sensitivity and general olfactory function

More than half (61.4%) of US older adults dwelling at home were able to detect androstadienone, ranging from 2.0% correctly detecting all four concentrations to 24.4% detecting only one out of four (Table 1). The remainder, 38.6%, however, failed to detect androstadienone at any concentration. Thus, the marked individual variation in androstadienone sensitivity and high rate of specific anosmia seen in young adults [24,29,71] is sustained in older adults, replicating Study 1. Women were more sensitive to androstadienone than men (p < 0.001) and sensitivity in both genders dropped with age (p < 0.001).

As expected from the laboratory study (Study 1), older adults were more sensitive to *n*-butanol, a standard physical odorant, than androstadienone, a social odorant. Fully 71.4% detected *n*-butanol in three or more pens, whereas only 8.8% failed to detect *n*-butanol at any concentration. (Table 1). A comparable share of older adults also had intact olfactory identification, correctly identifying 4 or more of the 5 different odors presented (77.6%).

*N*-butanol odor sensitivity was weakly correlated with androstadienone sensitivity in older U. S. adults (*r* = 0.19, p < 0.01) and remained when corrected for age and gender (*r* = 0.18, p < 0.01) as well as age, gender, and cognition (*r* = 0.17, p < 0.01). Odor identification was also weakly correlated with androstadienone sensitivity (*r* = 0.18, p < 0.01) and remained when corrected for age and gender (*r* = 0.14, p < 0.01) as well as age, gender, and cognition (*r* = 0.13, p <0.01). This significant positive association with general olfactory function had not been found in previous laboratory studies [29]. The current positive finding may have been enabled by the large sample size of a diverse population.

#### Social and sexual behavior

Older adults with better androstadienone sensitivity had more social connections and sexual behaviors during their everyday lives than those with low sensitivity or anosmia. Specifically, those with greater androstadienone sensitivity had more friends (p = 0.02), more close friends or family with whom they spoke at least once a year (p < 0.01) and participated more often in organized social events (p = 0.04; Table 3A, controlling for age and gender). Androstadienone sensitivity was not associated with how often respondents socialized with friends or family (p = 0.39).

**Table 3:** Associations between androstadienone sensitivity and social and sexual behaviors of older US adults living at home. **A**. **B**. Normosmic US older adults living at home: Associations between androstadienone sensitivity and social and sexual behaviors. (NB Tables are large and at the end of the manuscript).

	**Sample**	**US Population of Older Adults**		
**Social and Sexual Behaviors** ^**a**^	**N**	**Coefficient** ^**b**^	**95% CI**	**p-value** ^**c**^
# of friends	2,046	0.06	(0.01, 0.12)	**0.02‡**
# of close friends/family talked with ≥ once a year	2,081	0.09	(0.03, 0.15)	**<0.001‡**
Frequency of socializing with friends and family	1,821	0.03	(-0.03, 0.08)	0.34
Attending organized groups	1,813	0.06	(0.004, 0.11)	**0.04‡**
Volunteering	1,815	0.04	(-0.01, 0.10)	0.11
Had sex within the last 3 months	2,086	0.06	(0.01, 0.12)	**0.01‡**
Frequency of sexual thoughts	1,990	0.05	(0.01, 0.09)	**0.03‡**
Importance of sex	1,746	0.05	(-0.008, 0.11)	0.09†
Has an intimate partner	2,086	0.01	(-0.02, 0.03)	0.52
**Social and Sexual Behaviors** ^**a**^	**N**	**Coefficient** ^**b**^	**95% CI**	**p-value** ^**c**^
# of friends	1,534	0.07	(0.01, 0.13)	**0.03‡**
# of close friends/family talked with ≥ once a year	1,554	0.08	(0.01, 0.14)	**0.02‡**
Frequency of socializing with friends and family	1,381	0.04	(-0.02, 0.10)	0.21
Attending organized groups	1,374	0.06	(0.002, 0.12)	**0.04‡**
Volunteering	1,375	0.07	(0.004, 0.13)	**0.04‡**
Had sex within the last 3 months	1,556	0.07	(0.02, 0.12)	**0.01‡**
Frequency of sexual thoughts	1,490	0.06	(0.003, 0.11)	**0.04‡**
Importance of sex	1,328	0.06	(0.0001, 0.12)	**0.05‡**
Has an intimate partner	1,556	0.02	(-0.01, 0.04)	0.28

^a^ All models are linear regressions adjusted for age and gender.

^b^ Coefficients are standardized (mean = 0 and SD = 1). This means the coefficient is the correlation between androstadienone detection score and a given dependent variable.

^c^**‡ p ≤ 0.05,** † p ≤ 0.10.

Older adults with better androstadienone sensitivity were also more likely to have had sex recently (p = 0.01), had more sexual thoughts (p = 0.03), and tended to place greater importance on sex (p = 0.09). However, androstadienone sensitivity was not associated with being married or living with an intimate partner (p = 0.52, Table 3A), and thus the associations with sexual behavior (frequency, thoughts, and importance) were not simply an effect of being married or living with an intimate partner. Further, being married (or living with an intimate partner) did not alter any of the associations between androstadienone sensitivity and sexual behavior when it was included as a covariate.

The associations between social and sexual behavior and androstadienone sensitivity were driven in part by people with low androstadienone sensitivity or an anosmia. To ensure that the associations did not result from having poor olfactory function in general, we selected only those respondents who were normosmic (4 or 5 correct on the odor identification task) [33,54] and re-estimated all associations. Not only were all the previous associations still statistically significant in these “best” smellers (Table 3B), androstadienone sensitivity was also significantly associated with more frequent volunteering (p = 0.04) and reporting a greater importance of sex (p = 0.05; Table 3B).

Women were more likely than men to detect androstadienone odor (p<0.001 from unadjusted linear regression; S1 Table) and had more social and sexual behaviors associated with their androstadienone sensitivity than did men (four of five social behaviors in women vs. one in men and, including trends, all three sexual behaviors in women vs. one in men; Table 4A and 4B).

**Table 4:** A. Women: Associations between androstadienone sensitivity and their social and sexual behaviors. **B**. Men: Associations between androstadienone sensitivity and their social and sexual behaviors. (NB Tables are large and at the end of the manuscript).

	**Sample**	**US Population of Older Women**		
**Social and Sexual Behaviors** ^**a**^	**N**	**Coefficient** ^**b**^	**95% CI**	**p-value** ^**c**^
# of friends	1,080	0.12	(0.06, 0.18)	**<0.001‡**
# of close friends/family talked with ≥ once a year	1,098	0.08	(0.02, 0.15)	**0.02‡**
Frequency of socializing with family and friends	968	0.06	(0.002, 0.11)	**0.04‡**
Attending organized groups	965	0.10	(0.04, 0.17)	**<0.001‡**
Volunteering	966	0.05	(-0.03, 0.13)	0.19
Had sex within the last 3 months	1,100	0.06	(-0.01, 0.13)	0.10†
Frequency of sexual thoughts	1,048	0.07	(0.01, 0.13)	**0.02‡**
Importance of sex	929	0.07	(-0.006, 0.15)	0.07†
Has an intimate partner	1,100	0.03	(-0.007, 0.06)	0.12
	**Sample**	**US Population of Older Men**		
**Social and Sexual Behaviors** ^**a**^	**N**	**Coefficient** ^**b**^	**95% CI**	**p-value** ^**c**^
# of friends	966	-0.001	(-0.09, 0.09)	0.98
# of close friends/family talked with ≥ once a year	983	0.10	(0.004, 0.20)	**0.04‡**
Frequency of socializing with family and friends	853	-0.01	(-0.10, 0.07)	0.79
Attending organized groups	848	-0.003	(-0.08, 0.08)	0.95
Volunteering	849	0.04	(-0.04, 0.12)	0.37
Had sex within the last 3 months	986	0.07	(-0.01, 0.15)	0.09†
Frequency of sexual thoughts	942	0.02	(-0.05, 0.09)	0.61
Importance of sex	817	0.03	(-0.06, 0.13)	0.52
Has an intimate partner	986	-0.01	(-0.04, 0.02)	0.39

^a^ All models are linear regressions adjusted for age.

^b^ Coefficients are standardized (mean = 0 and SD = 1). This means the coefficient is the correlation between androstadienone detection score and a given dependent variable.

^c^ **‡ p ≤ 0.05,** † p ≤ 0.10.

Finally, we tested associations between androstadienone and variables that might confound or mediate its significant association with social and sexual behavior, each independently tested via unadjusted linear regression. Whites were more sensitive to androstadienone than African Americans (p < 0.001), contrasting with prior work with young adults (e.g. 20–30’s) in a convenience sample of New York City in which young African Americans perceived androstadienone as more intense than did other groups [72]. As expected, those with worse cognition had worse androstadienone sensitivity (β = 0.03, p < 0.001), as did those with worse self-rated physical health (β = 0.07, p < 0.01) although not those with low self-rated mental health (β = 0.03, p = 0.23) (all S1 Table).

However, none of these covariates (race/ethnicity, cognitive function nor self-rated physical health) explained the associations between androstadienone sensitivity and social or sexual behaviors. When each was added separately as a covariate to a significant model for normosmics (Table 3B controlling for age and gender), the coefficients remained essentially the same (difference between coefficients ranged from 0.00 to 0.02, S2 Table). We note that in some models, there was a slight decrease in the size of the coefficient, with corresponding p-values < 0.10 or a bit higher. This lack of substantial change indicates that none of these variables sufficiently explained the association between androstadienone sensitivity and social and sexual behaviors.

## General discussion

Androstadienone showed a trend toward biasing attention of older adults towards emotional faces that they could not consciously detect (p = 0.08), as it does in younger adults [24]. It specifically modulated attention to emotional information and not simply to another human, nor did it heighten attention in general. Rather, it selectively increased attention to emotional faces, an effect that persists throughout adulthood to old age. Presumably it operates by increasing functional connectivity between the amygdala, prefrontal and orbitofrontal cortical structures and visual cortex during visual emotional processing in older adults as it does in young adults [25].

The older adults who had their attention to subliminal emotional stimuli enhanced by androstadienone were also able to detect its odor (p = 0.05). This link between responding to androstadienone with enhanced emotional processing as well as detecting its odor likely rests on using the same androstadienone olfactory receptors, which have considerable allelic variation (e.g., OR7D4) [30]. Therefore, in our national field survey, we could use sensitivity to the odor of androstadienone as a proxy for its effects on emotional processing, attention and mood, which was demonstrated in controlled laboratory experiments [2,4,5,7,13,15,24,25,29,73]. While this study had a small sample size, its results, especially regarding androstadienone sensitivity, were validated in a much larger national sample in our field study.

Our studies in both the laboratory and the field confirmed striking individual differences in sensitivity to androstadienone odor. We extended prior findings in young adults in a homogenous Scandinavian population and samples of convenience in New York City [71,74] to the diverse population of older US adults, demonstrating that androstadienone sensitivity persists over the lifespan even while general olfactory function declines with age [75]. This wide variation is greater than that of most other odorants. In fact, androstadienone and androstenone (a structurally similar human steroid) had the widest variability in sensitivity of the 66 odorants examined in the New York City sample and was associated with the functional RT allele for OR7D4, encoding the receptor for androstadienone and androstenone [30]. Given these results, it is likely that these same olfactory receptor alleles are involved in both conscious and unconscious processing of compounds such as androstadienone, with concentrations needed for conscious odor perception being higher than the smaller concentrations sufficient for its unconscious or subliminal processing and subsequent effects on emotional attention and sexual behavior. However, the possibility remains that there may be an additional set of olfactory receptors involved in odor detection, with a smaller common set necessary for both conscious and unconscious processing. Further work may illuminate this by examining the connections between olfactory receptor genotype and social/sexual behaviors, moving beyond the link between androstadienone sensitivity and behavior to the mechanisms for such behavioral changes.

Exquisite attention to emotional information, either subliminal or conscious, contributes to social and sexual interactions in everyday life. Indeed, people who were more sensitive to androstadienone odor, and benefitted from having their attentional resources allocated to emotions, had richer social behavior and interactions during their daily lives, presumably benefitting from having more of their attentional resources focused on emotions. They had more friends, communicated more with close friends, attended organized events more frequently and volunteered more often. Whether this enhanced sociability results from better understanding of the emotions of friends, family, or strangers, including their fleeting microexpressions, awaits further study. The results do support the hypothesis that androstadienone plays a broader role in human social function across adulthood and is not limited to the sexual perceptions and sensuality of younger adults.

In addition, we report the first associations between androstadienone sensitivity and sexual behavior measured in everyday life, a social domain in which emotions play an important role. Older US adults with better androstadienone sensitivity had recently had sex more frequently as well as more sexual thoughts, viewing sex as an important part of their lives. It did not predict, however, the likelihood of being married or living with an intimate partner: 70% of US older adults had an intimate partner, of whom 96% of were married. This absence of an association may reflect the many reasons for marriage and intimate partnership beyond sexual intimacy in older adults that would override sensitivity to emotions. People may choose to be partnered due to monetary concerns or societal expectations, particularly in this generation when divorce was not common.

Along with being more sensitive to androstadienone’s odor, older women showed more significant associations with social and sexual behavior than did men, including more frequent socializing with friends and family. Nonetheless, men who were more sensitive to androstadienone still reported talking with more close friends and a trend towards more frequent sex in the past 3 months as did women. Many controlled laboratory studies of androstadienone have not detected sex differences in its effects on mood, emotional attention, or neural processing, although some have [6,73]. Future work will determine whether these disparities arise from small sample sizes, the artificial social context of laboratory and neuroimaging conditions, or sex differences in emotional responses to the same everyday life interactions [73,76–79].

While the magnitude of these relationships was small, they are nonetheless remarkable and not unexpected. First and foremost, our hypothesis that androstadienone may act as a broad social modulator, tuning attention to emotional information, is predicated on the idea that these changes occur in a subtle and subliminal fashion. In this sense, one might expect relatively small associations with actual behavior in everyday life. Human beings are complex social organisms and it is reasonable to expect that the effects of chemosignals like androstadienone would have subtle modulatory effects on our behavior, in contrast to the classical pheromones that elicit “definite behaviour” in lower organisms [80]. Second, sociosexual behaviors were not measured at the time of androstadienone exposure. Instead, they were reported retrospectively as typical patterns throughout a long-time frame (months to years) while sensitivity to androstadienone odor was measured only once during 5 minutes of the NSHAP interview. Nonetheless, their associations were detectable. Third, androstadienone sensitivity was measured at only 4 concentrations in NSHAP, rather than the more comprehensive androstadienone sensitivity measures used in the laboratory (N = 16) and others (N = 5) [29]. Logistical constraints of the NSHAP interview mandated substantially shortening the sensitivity test [33]. As a result, we were forced to sacrifice some measurement reliability associated with more comprehensive methods that use a greater number of concentrations. That we are still able to identify these associations with such a modest measure of androstadienone sensitivity suggests the associations reported here may in fact be conservative.

General olfactory function declines with age, particularly among older adults [69,71,75,81], which might create a spurious association between androstadienone sensitivity and sociosexual behavior, particularly given the weak association between androstadienone sensitivity and general olfaction function. Worse olfactory function predicts worse physical and mental health [55,82] which in turn may independently reduce sociosexual behaviors.

Although androstadienone sensitivity had not been associated with overall olfactory acuity in small samples of young and older adults [29], with a large representative sample of older US adults we did detect a relationship between lower androstadienone sensitivity and both poor *n*-butanol sensitivity and odor identification.

Nonetheless, we determined that worse olfactory function did not confound our results by limiting our analyses to those who had normal olfactory function and finding that androstadienone sensitivity was still significantly associated with both social and sexual behaviors. The strength of all associations remained the same or increased when controlling for general olfactory function, with one exception that was still significant (talking more to those you feel close to).

Androstadienone is a modulatory pheromone, produced by both men and women, and affects the social and sexual lives of those adults with the capacity to respond to it. Presumably, it operates during everyday life of US older adults by enhancing emotional processing which facilitates social and sexual interactions. The full range of emotional information needs to be expanded, particularly to include negative emotions such as fear [83] as well as to other social modalities conveying emotion such as speech and words. Androstadienone also modulates a person’s mood and arousal states; that is, their positive and negative reactions to events in their lives during social situations in both the laboratory and during everyday life [4,5,13,15,18,84]. For example, androstadienone sustained positive moods during and after being in the laboratory and prevented a rise in negative moods [4,15]. Therefore, the range of behaviors effected during everyday life are not likely limited to those measured here.

As our laboratory study showed, androstadienone is an airborne vagile compound, not requiring direct contact with the skin (confirming the chemosensory route seen in young adults) [6,13,83]. It is also effective in nanomolar amounts below conscious detection as an odor, and its effects persist for up to 2 hours [4]. Individual differences in the capacity to detect and to respond to androstadienone may be meditated by the receptor OR7D4, but the sex differences in the association between odor sensitivity and sociosexual behavior suggest that other receptors may be involved, just as androstadienone is likely to be but one example of social chemosignals that modulate human emotions and behaviors.

## Supporting information

S1 Table Relationship between sex, age, race/ethnicity, cognitive function, physical health, and androstadienone sensitivity. ^a^ displaying coefficient^b^ (p-value^c^). (NB Supplementary Tables are found in the supplementary material).
(TIF)

S2 Table Testing potential confounders of the significant associations between androstadienone sensitivity and social and sexual behaviors of normosmics, controlling for age and gender ^a^(Column 1) and then adding each of the three variables one at a time (coefficient^b^, (p-value^c^)). (NB Supplementary Tables are found in the supplementary material).
(TIF)
