# Supplementary figures and images for: Androstadienone sensitivity is associated with attention to emotions, social interactions, and sexual behavior in older U.S. adults
Source: PLoS One. 2023 Jan 13;18(1):e0280082. doi: 10.1371/journal.pone.0280082 (PMC9838868; doi:10.1371/journal.pone.0280082)

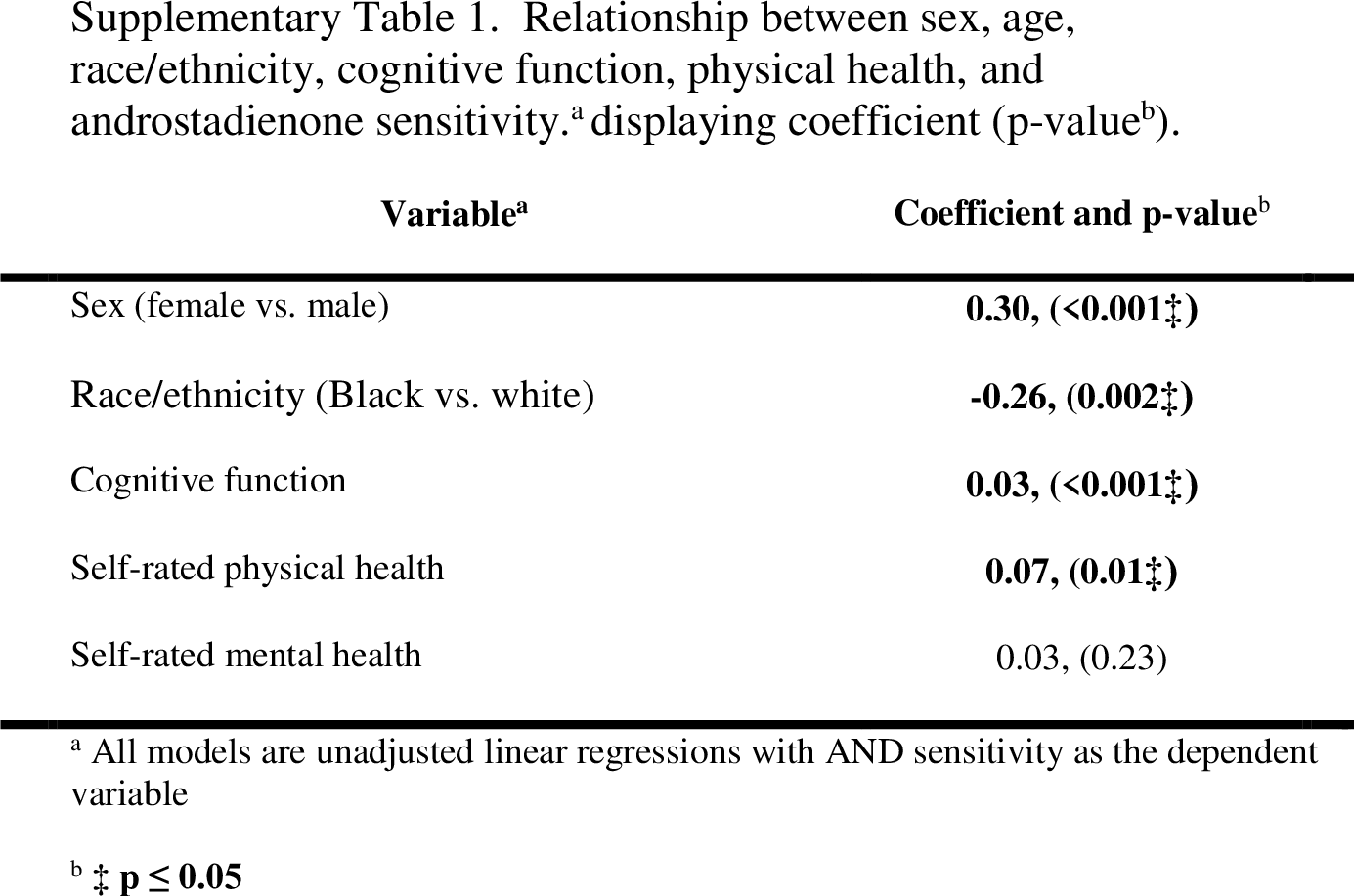

Supplement: S1 Table — a displaying coefficientb (p-valuec). (NB Supplementary Tables are found in the supplementary material). (TIF) [file pone.0280082.s001.tif]

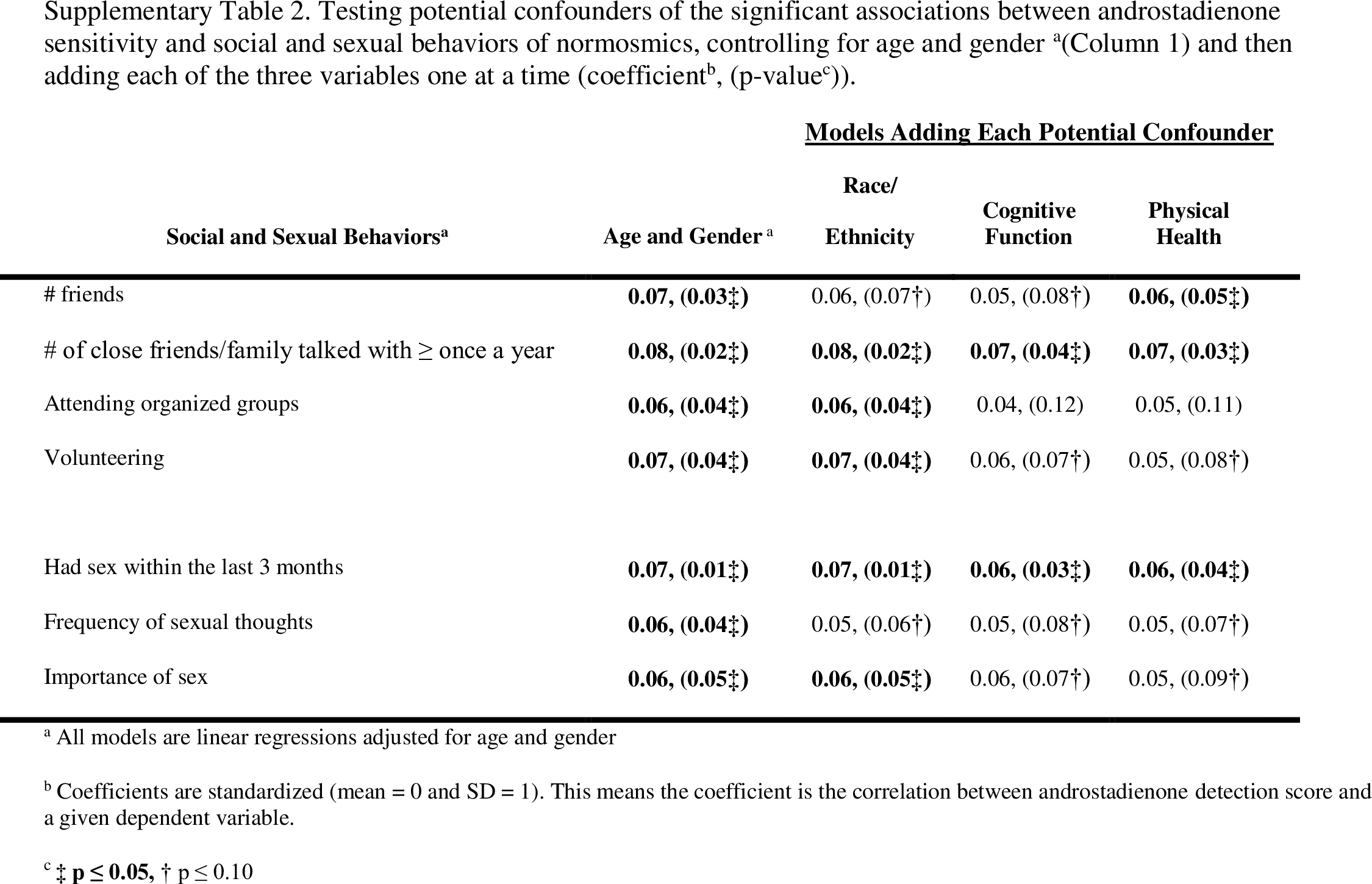

Supplement: S2 Table — (NB Supplementary Tables are found in the supplementary material). (TIF) [file pone.0280082.s002.tif]
